# Supplementary material for: Updating the remembered position of targets following passive lateral translation
Source: PLoS One. 2024 Dec 31;19(12):e0316469. doi: 10.1371/journal.pone.0316469 (PMC11687815; doi:10.1371/journal.pone.0316469)
Supplement: Attachment — Submitted filename: Review_KIM and HARRIS.docx [file pone.0316469.s001.docx]

Kim and Harris report that during lateral translation, subjects systematically underestimate target eccentricity, particularly for more eccentric targets, with errors influenced by whether the target crosses the observer's midline. These errors likely reflect changes in spatial updating tied to perceived self-motion and reconstructing target positions across visual fields. While the results are interesting, the manuscript would benefit from clearer data representation, both in the text and figures. Additionally, the writing (several errors) could be substantially improved (could be more compact) for better clarity and impact.

**Major issues:**

**EXPERIMENT 1**:

- The text is not clear. What is the spacing between targets? I agree that it is mentioned in the “methods’ but it is good to remind the reader of the relationships between target positions, travel range, and motion simulation. My major concern here is the overinterpretation of the main effect (page 15, lines 15-16) as reported by a significant interaction (motion and target) with p = 0.045. I suggest emphasizing the small effect size in the interaction, and a much clearer explanation for spatial updating. What are the data points to the extremes of these lines (- 0.75 and 0.75), though the translation is between – 0.5 and 0.5? Since all the subjects are right-handed, was there any hemi-field effect (it is reported that errors increased with eccentricity, it seems there is no hemifield effect but could be mentioned / investigated further)? Can the authors indicate the significance on the graph? I think it is more informative and intuitive to switch the x and y axis. The data should be plotted: Remembered target position as a function of actual target position. That way the cartoon on the right will be aligned on the x axis, thus better representing the horizontal translation (and motion). Along the same lines, instead of a columnar organization for the figure, the data could be represented in a row.
- This sentence is not clear (Page 15, lines 18-21): “In the Visual Motion condition (where sideways motion was simulated visually), the errors significantly differed (p < .001) between the targets at 0m and .25m (Targets at -0.5m and -0.25m in Fig 3A, Targets at 0.25m and 0.5m in Fig 3B).” Please clarify.
- The statement (lines 2-4, page 16) is vague: ‘suggesting that updating may be dependent on target eccentricity as well as the travel range since those targets within the range may be updated differently compared to those outside the range.’’ What does ‘differently’ mean?
- Accordingly, the spatial updating conclusion should be clearly derived. I agree that the platforms are being passively moved, given that the neck was restrained, the head did not contribute to the updating mechanism(s). Can the authors speculate in discussion how the head may contribute to it (thus reaching) in the real world?

**EXPERIMENT 2:**

- What led to Experiment 2 from Experiment 1? The text should begin with a reason for Experiment 2. What did the authors choose a different range (-.46 - .46) in this exp compared with Exp 1? Figure 5 seems to explain this but the decision for this range should be mentioned here too. It is also discussed in ‘discussion’. Despite that, it should be mentioned here.
- motion was 13 ±0.46m. Not clear. Is it motion (should be in m/s ) or initial position (as I see in table 1?)
- Why does Fig 4C precede 4A and 4B? The text should follow systematically with figures. I can see the effect of the target eccentricity on errors in Fig c, but the authors do not do justice to the figures by explaining them well. Furthermore, how do the authors explain the larger spread of errors during physical motion than the other two conditions? In fact, it also seems evident in 4C. I also suggest plotting these errors with all the data points.
- The authors mention (Page 18, line 1): “However, unlike in Exp 1, the errors did not differ between the motion conditions”. How do they exactly mean it? Any stats?
- Please look at my comments on EXP 1 for figures (you can go along those lines). The authors should describe visual (left), physical (middle) and full motion(left) better in the text. Again, the data can be plotted as suggested in 1. Why does the label change here to ‘Perceived target location’? Keep it consistent.

**EXPERIMENT 3:** See my earlier comments, however, to plot the difference between the short and long distances, the graph doesn’t help much. Can the authors think of any other way of plotting the data? Moreover, the tests they performed are rather opaque to the reader.

- Here in the text: Post hoc evaluation of paired t-test, with Holm-Bonferroni correction showed that the mean errors made for each target position significantly differed between motion conditions, where the errors shifted after moving compared to Stationary in the direction of travel for both short (p 20 = .039) and long (p < .001) travels. The errors also differed between the short and long travel 1 distances (p = .002), in which longer travel distances resulted in larger errors.
- How do confirm the above effect in both directions? Are the data pooled (normalized) for both directions? Please clarify.
- Can the authors plot the data (errors) in the spatiotemporal domain: errors as a function of the reaction time?
- I suggest moving the figures from discussion into the results section. This will help to further streamline the discussion section (to make it shorter).
- The authors could discuss a neural mechanism involving the integration of spatial information at the neural across brain areas. While Dash et al. (2015) [Continuous Updating of Visuospatial Memory in Superior Colliculus during Slow Eye Movements] focus on the gaze system, the authors might suggest something along these lines for the reaching system. In other words, how the gaze system may be involved in updating reaching plans, while also discussing perception of self-motion.

**Other issues:**

- Introduction: Needs to be streamlined (and could be more compact) with clear hypotheses. Should briefly mention results and conclusion.
- Discussion: Needs to be substantially shortened.
- Line 13: omit ‘of course’ and several other instances where the language is not scientific (e.g., obviously).
- Page 21, lines 14-15. These results could be mentioned in the results section.
- Remove contractions such as: ‘didn’t’ should be replaced with ‘did not’.
- What is the size of the virtual tennis ball?
- What does a passive movement mean? A little detail would be beneficial for the reader.
- Page 6, line 14: what is FOV?
- Make sure the units are represented consistently and with a ‘space’ between the value.
- Figure 2 (E): In the figure I see the background white rectangle. If this is the case, the participants can use it as an allocentric landmark. Did the experiments simulate darkness? I understand that there was some virtual background to the scene. What us the color of the fixation dot and other features during presentation?
- At several instances the text should read ‘compared with’ instead of ‘compared to’. These two have specific meanings.
