# Supplementary material for: Updating the remembered position of targets following passive lateral translation
Source: PLoS One. 2024 Dec 31;19(12):e0316469. doi: 10.1371/journal.pone.0316469 (PMC11687815; doi:10.1371/journal.pone.0316469)
Supplement: Attachment — Submitted filename: Response to Reviewers.docx [file pone.0316469.s003.docx]

Response to Reviewers:

**Reviewer #2**: The authors have made great efforts to address the comments.

I have a few minor points left:

1. The section on the updating ratio should be moved to the analysis (Methods) section - it sticks out in its current position.

- Revised.

2. While I appreciate that the authors are trying to highlight the similarities and differences in the procedure section for the three experiments, I would prefer a more detailed description for each experiment first, followed by a short section where similarities and differences are highlighted. This will make the interpretation of the results even easier.

- The procedure section now has 3 subsections to explain each experiment separately.

3. A summary paragraph at the end of the discussion section would be useful.

- We added a conclusion section after the discussion that summarize the main findings.

4. Some additional proofreading is required; though, these are very minor.

- Revised.

**Reviewer #3**: The authors have addressed all my comments and concerns and also provided further clarifications. This work significantly enhances our understanding of perceptual processes during self-motion especially during lateral translation. Good work.
